# Interface between obesity with dysfunctional metabolism and inflammation, and the triple-negative breast cancer in African American women

**DOI:** 10.37349/etat.2021.00066

**Published:** 2021-12-31

**Authors:** Katarzyna Rygiel

**Affiliations:** Department of Family Practice, Medical University of Silesia, 41-800 Katowice-Zabrze, Poland; University of Edinburgh, UK

**Keywords:** Triple-negative breast cancer, African American, body mass index, waist-to-hip ratio, central obesity, cytokines, inflammation, tumor microenvironment

## Abstract

Obesity has dramatically increased over the past fifty years. In the last decade, it has been noted that augmented body mass, metabolic abnormalities, and the relevant “obese” tumor microenvironment (TME) are connected with signaling molecular networks, which in turn, may contribute to aggressive tumor biology in some patients with breast malignancies. This article presents the associations between obesity, metabolic derangements, inflammatory processes in the adipose tissue or TME, and aggressive behavior of triple-negative breast cancer (TNBC) in African American (AA) women. It also describes some abnormal molecular signaling patterns in the “obese” TME with relevance to TNBC biology. Ethnic disparities in TNBC can be due to a variety of biological features (e.g., genetic mutations and tumor heterogeneity), comorbidities (e.g., cardio-metabolic diseases, including diabetes mellitus), and reproductive factors (e.g., multiparty or short breastfeeding period). Such a constellation of biological variables potentially leads to the association between obesity, metabolic derangements, inflammatory processes in the adipose tissue or TME, and aggressive behavior of TNBC in AA women. Since the TNBC and its TME can display very aggressive behavior, it is crucial that the afflicted AA women make efforts to maintain healthy body weight, “flexible” metabolism, and a well-functioning immune system. Further studies are merited to explore the multi-disciplinary factors that can affect TNBC prevention, management, and outcomes to optimize treatment strategies and survival among AA women.

## Introduction

Obesity has dramatically increased over the last fifty years, in several countries, worldwide. It should be underscored that its perception and interpretation have been transformed from a condition often related to general wealth to the one linked with low financial, social, or health status [1]. Obesity is a multi-factorial, serious medical condition related to genetic, metabolic, inflammatory, behavioral, and environmental factors. Obesity is defined as a body mass index (BMI) ≥ 30 kg/m^2^ [calculated as the body weight in kilograms (kg) divided by the square of height in meters (m)] [2]. BMI represents an easy and inexpensive screening method for categorizing weight (e.g., obesity, overweight, normal weight, and underweight). Although the BMI correlates, to some degree, with the total body adipose (fat) tissue content and the relevant metabolic abnormalities or comorbidities, it is often inadequate to evaluate a person’s metabolic health, especially in ethnic minorities [1, 2].

Traditionally recognized body shapes of obesity include the “apple” or android, upper body (also known as an abdominal or central) obesity (with visceral fat reservoirs located at the waist level) and “pear” or gynoid, lower body obesity (with fat deposits on the thighs and buttocks), which are in association with inflammation, abnormal metabolism, and breast cancer (BC), playing an important role in evaluating cardio-metabolic and neoplastic risks. A simple anthropometric parameter, such as waist-to-hip ratio (WHR) [which is computed as waist circumference (WC) divided by hip circumference] is helpful in an assessment of abdominal obesity. WHR ≥ 0.85 is a risk factor for metabolic and inflammatory complications of obesity, and a higher WHR is associated with a large spectrum of metabolic and hormonal derangements that can serve as a possible predictor of the triple-negative breast cancer (TNBC) risk [3]. Such simple anthropometric parameters of body shape (e.g., WC or WHR), which are useful as practical tools, detecting cardio-metabolic and neoplastic risks, are mostly measuring central obesity [3, 4]. However, these anthropometric assessments may not be accurate, especially in some cases, in which the metabolism, inflammation, and BMI appear to be poorly associated [3].

Since central obesity with related abnormal metabolism increases tissue inflammation via the production of cytokines and growth factors, it can also aggravate local invasiveness and distant metastases of TNBC, contributing to worse outcomes, especially in the African American (AA) women patient population [4]. In fact, insulin resistance (IR) is one of the causes that may lead to a worse BC prognosis among AA *vs.* European American (EA) women [4]. IR and hyperinsulinemia are the main components, which underlie the development of the metabolic syndrome (MS) that is defined as the presence of at least three of the five criteria: 1) WC ≥ 88 cm, 2) triglycerides ≥ 150 mg/dL (or on therapy for hypertriglyceridemia), 3) high-density lipoprotein < 50 mg/dL, 4) fasting glucose level ≥ 100 mg/dL, and 5) systolic blood pressure ≥ 130 mmHg or diastolic blood pressure ≥ 85 mmHg (or on treatment for arterial hypertension) [4]. Moreover, it is noted that hyperinsulinemia can contribute to the growth and metastatic spread of BC via activation of the insulin receptor/insulin-like growth factor 1 receptor (IGF-1R) signaling pathways [4].

In addition, obesity and its associated poorly controlled comorbidities [e.g., diabetes mellitus type 2 (DMT2), arterial hypertension (HTN), or cardiovascular (CV) disease] can further complicate or delay effective TNBC management [4]. In fact, these comorbidities are often accompanied by increased chronic inflammation on both systemic (evaluated via biomarkers in the blood) and local levels (in fat tissue reservoirs) [3, 4]. Moreover, this chronic inflammation is characterized by disequilibrium of T cells and cytokines, which can adversely influence the tumor microenvironment (TME) of patients with BC. It should be underscored that some obese women, with high BMI but functional metabolism and low degree of inflammation, seem to have a decreased risk for some obesity-related malignancies. On the other hand, some normal weight or just slightly overweight women, which are with dysfunctional metabolism and increased inflammation, may suffer from augmented BC risk (particularly among ethnic minorities). This implies a necessity of conducting future prospective clinical trials, which will help identify the women with “hidden” risks of “obesity-associated” BC [3, 4].

**Figure 1.**
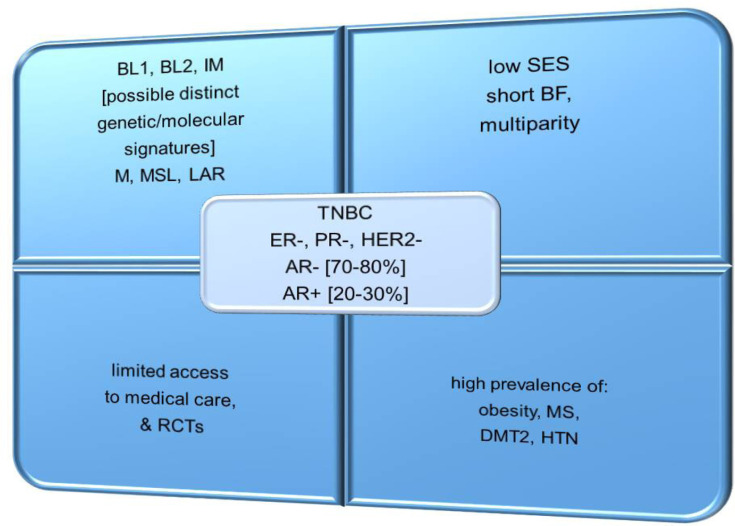
Common features of TNBC in AA *vs.* EA women. IM: immunomodulatory; M: mesenchymal; MSL: mesenchymal stem-like; BF: breastfeeding; RCTs: randomized controlled trials; SES: socio-economic status

TNBC is an aggressive subtype of BC, characterized by an absent or very low expression of estrogen receptor (ER), progesterone receptor (PR), and human epidermal growth factor receptor 2 (HER2) [5]. In addition, about 20–30% of TNBCs are androgen receptor (AR)-positive, and display a luminal androgen receptor (LAR) subtype that is characterized by lower sensitivity to standard chemotherapy (CHT) and a low proliferation rate [5]. In contrast, approximately 70–80% of TNBCs are AR-negative, which usually have a greater sensitivity to CHT and a higher proliferation rate [5]. TNBC is classified as basal-like (BL1 and BL2), immunomodulatory, mesenchymal, mesenchymal stem-like, and LAR subtypes, which are regulated by various signaling networks (Figure 1) [5, 6]. When comparing different ethnic populations of women from various geographical locations, it is remarkable that the incidence of TNBC is higher in West African and AA women than in EA women [6]. These intriguing differences may indicate certain genetic predispositions to TNBC among women of African descent [6]. 

In addition, it is still undetermined whether some differences exist in the inflammatory gene expression between upper body and lower body subcutaneous fat tissue in black *vs.* white women [7]. According to a small clinical study, black women had higher inflammatory gene expression levels compared to white women, but the relationship between fat tissue inflammation and insulin sensitivity was stronger in white than in the black group of females [7]. Somewhat unexpectedly, gluteal subcutaneous fat tissue revealed a higher inflammatory gene expression compared to the abdominal fat tissue reservoirs [7]. However, more research is needed to elucidate the role of upper body and lower body subcutaneous fat tissue depots and different levels of expression of inflammatory cytokines or macrophage (M) markers among black women [7]. It should be underlined that ethnic differences in inflammatory cytokine [e.g., interleukin-6 (IL-6) and interferon-gamma] levels and their correlations with abnormal metabolism can provide some basis for existing disparities in BC between AA and EA women, pending additional studies in this area [8]. For this reason, monitoring the inflammatory responses may help with lifestyle modification interventions to decrease BC risk, especially in minority ethnic populations [8].

This article presents the associations between obesity, metabolic derangements, inflammatory processes in the adipose tissue or TME, and aggressive behavior of TNBC in AA women. It also describes some abnormal molecular signaling patterns in the “obese” TME with relevance to TNBC biology. Finally, it outlines some strategies related to weight management (such as lifestyle modification, including healthy eating, physical activity, psychological education, and support), which should be helpful to prevent or reduce, to some degree, the occurrence, recurrence, and mortality due to TNBC among AA women.

## Multi-level connections between obesity, inflammation, and TNBC in AA women–remarkable lessons learned from the African American Breast Cancer Epidemiology Risk Consortium epidemiologic studies

There are complex multi-level interrelations between the body anthropometric parameters, metabolic, hormonal as well as inflammatory factors and the risk of TNBC [3, 9]. Moreover, TNBC may have stronger connections with various components of the MS (e.g., central obesity, IR, impaired glucose tolerance or DMT2, dyslipidemia, and HTN) than those with the excessive production of hormones [3, 9].

To explore these topics in more depth and to address relevant ethnic differences, the African American Breast Cancer Epidemiology and Risk (AMBER) Consortium project has investigated the discrepancies among results observed within the individual studies (e.g., regarding the correlations between obesity measured either by BMI or WHR and TNBC) [10]. The AMBER Consortium includes the Carolina Breast Cancer Study (CBCS) [11], the Women’s Circle of Health Study (WCHS) [12], the Black Women’s Health Study, and the Multiethnic Cohort Study [10].

The AMBER Consortium studies reveal that in general, among premenopausal females, the elevated BMI is rather associated with a lower incidence of all BC subtypes (including TNBC) [10]. However, the TNBC risk is predominantly increased among younger or premenopausal AA women that commonly suffer from central obesity [10]. In addition, for premenopausal women, an increased WHR is often related to an elevated risk of premenopausal ER-positive breast tumors and all subtypes of BC that usually occur in the postmenopausal stage of life [10]. However, for AA women, some different mechanisms that are responsible for the associations between adiposity (e.g., central), TNBC, or ER-positive BC are probably involved [10]. Unfortunately, anthropometrics may not be adequate to predict the exact level of metabolic or hormonal functioning. For instance, some women can suffer from obesity, but they may not have metabolic dysfunctions such as dysglycemia and dyslipidemia. In contrast, there are many females who may have an appropriate body mass, but they still can suffer from metabolic abnormalities (e.g., hyperglycemia or hypercholesterolemia). Moreover, the ability of BMI to predict metabolic status can differ among individual women (e.g., depending on their ethnicity, age, comorbidities, or menopausal status) [10]. The results of the AMBER Consortium studies suggest that the impact of general and central obesity varies by menopausal status and hormone receptor (HR) types among AA women. Also, it is suggested that mechanisms which link adiposity with the TNBC or ER-positive BC differ considerably across ethnic groups of patients [10].

The CBCS was a large case-control study of biological and social risk factors for invasive BC. Importantly, a selection of cases was population-based and stratified by ancestry and age at BC diagnosis, while controls were matched to the cases by age, ethnicity, and neighborhood [11]. According to the CBCS, elevated values of BMI and WHR augment the likelihood of basal-like TNBC in premenopausal AA females [11]. Also, based on the CBCS, it was found that obesity (defined by an increased WHR) was linked to an increased incidence of TNBC in premenopausal and postmenopausal AA women [11]. Moreover, based on the sequencing genomic DNA (from 1,370 cases and 1,635 controls), approximately 5.6% of patients are the carriers of a pathogenic variant in breast cancer gene 1 (*BRCA1*), *BRCA2*, partner and localizer of *BRCA2* (*PALB2*), or tumor protein 53 which are the main penetrant genes of BC [11]. Notably, in the CBCS, the relation between TNBC and *BRCA1* (*vs.* other BC subtypes) was shown [11]. These findings also indicate a possible association between TNBC (*vs.* other BC subtypes) and *BRCA2* or *PALB2* among AA women instead of EA women [11]. Moreover, AA patients with pathogenic variants in *BRCA2* or *PALB2* are over ten times more likely to be diagnosed with TNBC (*vs.* other BC subtypes) than EA patients who had pathogenic variants in either of these genes. If this pattern is confirmed in further studies, it might be expected to explain the increased prevalence of TNBC in AA patients to some degree [11].

Similarly, the WCHS which was designed as a multisite case-control study in the US attempted to decipher the potential risk factors for early aggressive BC among AA and EA women [12]. The WCHS findings suggest that BMI is generally unrelated to BC in AA women [12]. However, higher waist and hip circumferences and an increased WHR are associated with increased premenopausal BC risk, while general obesity is related to decreased risk of ER-negative/PR-negative BC subtypes [12]. Nevertheless, in the future, more studies are needed to confirm such findings and to further evaluate the impact of obesity and body fat distribution on various BC subtypes in different ethnic groups of women.

Based on a study comparing biomarkers associated with obesity with abnormal metabolism among AA women (with and without BC), it was shown that the AA women with BC revealed higher IR as well as elevated biomarkers of dyslipidemia (e.g., cholesterol and triglyceride fractions) and inflammation [e.g., IL-1b, IL-6, IL-8, tumor necrosis factor-alpha (TNF-alpha), and C-reactive protein (CRP)] compared to the age-similar controls (or even much older females) [13]. Such biomarkers of inflammation and IR could be related to an elevated risk of BC recurrence, and thus continuous monitoring of this group of AA women would be merited [13].

Furthermore, it should be highlighted that the gene expression evaluation (e.g., BC subtype- and stage-specific analysis) based on the BC data from The Cancer Genome Atlas reveals that the numbers of differentially expressed genes between AA and EA patients increase in every stage of BC progression [14]. This may indicate an increased genomic instability that accompanies BC progression. For instance, *resistin*, a gene that is linked to obesity, IR, DMT2, and BC, shows an amplified expression in BCs of AA patients. Also, a long and non-coding RNA LOC90784 is downregulated in tumors of AA patients, and its expression is inversely related to the BC stage (e.g., a loss of the expression of LOC90784, especially in TNBC, could contribute to adverse outcomes in AA women with TNBC) [14]. In addition, an elevated expression of *p53* and *BRCA1* networks is noted in samples of breast tumors obtained from AA patients [14]. Hopefully, gaining a deeper insight into molecular mechanisms which may drive disparities in BC will help detect some novel biomarkers and relevant treatment targets (e.g., *resistin*, or the aurora B and polo-like kinase signaling pathways) for more personalized care of certain AA patients with BC [14].

A recent population-based longitudinal study of AA survivors of BC provides some valuable data that may allow determination of a possible contribution of the genetic, epigenetic, and molecular mechanisms, CV, or metabolic factors in concert with environmental toxicities and social or behavioral components to poor outcomes and elevated death rates in AA women with BC [15]. This type of research should help create effective strategies to overcome disparities in BC care among AA women in the future [15].

In addition, some important differences between AA and EA women based on their body shape (obtained via digital photos), adipose tissue distribution (defined by the android-gynoid ratio), and body composition (assessed with the dual-energy X-ray absorptiometry application) are reported [16]. Results of this study show that AA women had a higher BMI, android-gynoid ratio, total body fat, trunk fat, and leg fat in comparison to EA women [16]. Also, the “apple” body shape is more prevalent in AA than that in EA women [16]. It appears that the obese AA and EA women exhibit some differences not only in their distribution of adipose tissue but also with regard to the metabolic parameters (e.g., IR or lipid fraction levels), as well as predispositions towards certain BC subtypes [16]. However, further studies are needed to confirm these findings.

## A transition from normal anthropometric and metabolic parameters to obesity, dysfunctional metabolism, and pro-inflammatory status

**Figure 2.**
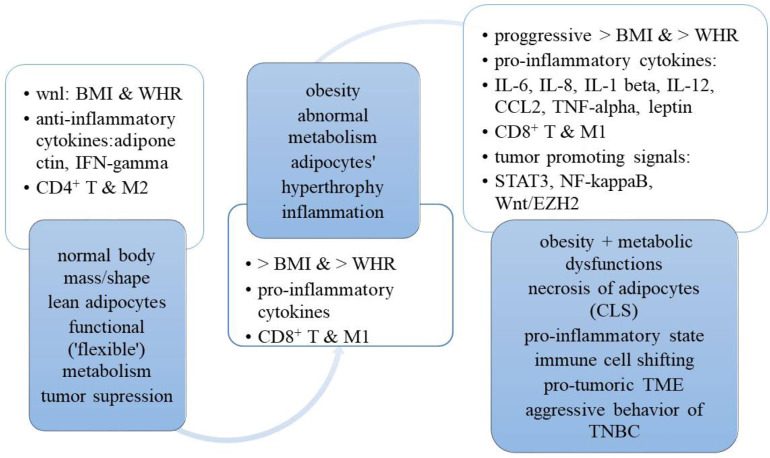
A transition from normal body mass/shape and metabolism to obesity, dysfunctional metabolism and pro-inflammatory/pro-tumoric “milieu” for TNBC. CCL2: chemokine (C-C motif) ligand 2; CLS: crown-like structures; wnl: within normal limits

It has been suggested that M and T cells can play a dual role via blocking or enhancing the aggressive behavior of TNBC. In particular, activated M1 macrophages are regulated by Th1 cytokines [e.g., interferon-gamma (INF-gamma) or TNF-alpha]. INF-gamma is an effector cytokine with anti-proliferative, pro-apoptotic, and antitumor mechanisms. TNF-alpha is an inflammatory cytokine (produced by macrophages or monocytes during acute inflammation) that regulates signaling in cells (that leads to necrosis or apoptosis) and is responsible for resistance to pathogens and carcinogens. When M1s exhibit increased cytotoxicity, they perform anti-tumorigenic actions [17]. However, if tissues are affected by pro-inflammatory cytokines (e.g., IL-6, IL-8, IL-12, IL-1beta, and leptin), there is a shift from M1 to M2 macrophages [18]. Unfortunately, M2 macrophages that are often present in the tumor stroma enhance the malignant process and deteriorate the prognosis of TNBC (Figure 2) [19].

It is important to realize that there is a transition period from normal anthropometric and metabolic parameters to obesity and dysfunctional metabolism, which is associated with a production of pro-inflammatory cytokines which can aggravate a pro-tumorigenic milieu in the tissues. On the one hand, this time period can be considered as a “prelude” to cancer. On the other hand, however, this dangerous situation also creates a possible “window of opportunity” for women at risk for TNBC. For instance, it would be beneficial to effectively intervene within the early stages of this unfavorable biologic path. In particular, a proactive, non-invasive intervention via lifestyle modification (e.g., healthy nutrition, regular physical exercises, educational courses, supportive services, and stress reduction) may possibly redirect, to some degree, this dangerous trajectory towards an appropriate body mass and functional metabolism (e.g., carbohydrates and lipids). This, in turn, may restore proportions between anti- and pro-inflammatory cytokines and reinforce immunologic defenses. A combination of easily accessible anthropometric (e.g., BMI and WHR) and metabolic parameters [e.g., lipid fraction profile and hemoglobin (Hb) A1C], as well as some pro-inflammatory biomarkers (e.g., CRP, leptin/adiponectin ratio, or IL-6/IL-8 index) may bring valuable information about a potential TNBC biologic behavior [17]. In order to promptly detect patients who are at risk for the most aggressive TNBC behavior, the assessment of BMI and WHR, as well as laboratory tests (e.g., lipid panel, HbA1C, and CRP) appear feasible and should be done whenever it is possible (e.g., in primary care setting).

## A route to a pro-tumorigenic milieu for TNBC—a role of pro-inflammatory cytokines in deteriorating the immune defenses

It is important to realize that when the anthropometric body parameters are within normal limits and adipose tissue is lean, the M2 macrophages (M2) together with the T regulatory cells are keeping the inflammatory processes within the adipose tissue [17]. Such a balance in metabolic and immune regulation can be achieved via IL-4 and IL-13 (originating from eosinophils), as well as IL-10 (originating from T regulatory and M2) [17]. This allows limiting the inflammatory processes to local areas [17]. However, it should be underscored that some harmful dietary components (e.g., saturated free fatty acids) often consumed by women with obesity can promote the production of pro-inflammatory IL-1 beta, which enhance the M1 recruitment to the adipose cells. Under these circumstances, M1s secrete pro-inflammatory cytokines (e.g., IL-6, IL-8, IL-12, IL-1 beta, and TNF-alpha) which can escalate inflammation, manufacture necrotic lesions in adipocytes (e.g., crown-like structures), and contribute to the inflow of the effector and memory T cells. This, in turn, can initiate or create a “milieu” that is conducive to aggressive TNBC behavior of the afflicted patients (Figure 2) [17].

## An exploration of ethnic differences in BC risks between AA and EA—addressing some new research directions in the tumor microenvironment and immune responses

It should be underscored that the inflammatory immune response is stronger in AA women than that in EA women [20]. Traditionally, HR-negative BCs are considered as immunologically “hot”, while the HR-positive ones—as immunologically “cold” tumors. In fact, the ethnic differences in the immune system can also be related to the TME, which may be partially responsible for the BC aggressive biology, often leading to worse outcomes in AA patients [12, 20]. For instance, when comparing some ethnic differences in breast tumor immune phenotypes, it is found that there are important discrepancies in the tumor immune responses between AA and EA women. In particular, AA patients display a stronger CD4^+^ and B-cell response, and their breast tumors are characterized by larger immune infiltrates (regardless of BC subtypes) [20]. However, in the breast tumors from AA patients, a more exhausted CD8^+^ profile is observed [20].

Such a pattern of the higher proportion of exhausted CD8^+^ T cells to total CD8^+^ T cells is related to worse survival, especially in HR-positive patients with BC. Also, in the HR-negative patients, it was noted that combinations of the absolute fraction of CD8^+^ T cells and the exhausted CD8^+^ T cells to total CD8^+^ T cells allowed detection of the subgroup, which was characterized by the highest proportion of the CD8^+^ low and exhausted CD8^+^ T cells to total CD8^+^ T cells. This subgroup was more prevalent in AA women, and these patients had the worst survival [20]. Also, in the HR-negative patients, the subgroup characterized by the highest proportion of the CD8^+^ low and exhausted CD8^+^ T cells to total CD8^+^ T cells was detected [20]. It was noted this subgroup was more prevalent in AA women, and these patients had the worst survival [20].

These results suggest that there is an immunobiological reason for a more aggressive BC behavior in AA women. Such findings may create a realistic hope for a possible therapeutic targeting of the exhausted immune phenotype in AA patients with the application of immune checkpoint inhibitors [20]. However, the patient responses have not yet been fully evaluated as a function of obesity and metabolic complications, and thus, future studies in this area would be merited.

## A contribution of SES to the disparities in TNBC outcomes in AA women

In addition to the above-mentioned biologic factors, it should be highlighted that some serious obstacles in the access to healthcare, mostly due to a low SES that often contributes to disparities in the BC outcomes [21]. In particular, low SES is associated with an increased risk of aggressive premenopausal BC, late-stage diagnosis, and decreased survival in AA women [21, 22]. For instance, the role of ethnicity in the survival of women with TNBC was examined based on the medical records of patients from a hospital in Louisiana (one of the Southern US states, densely inhabited by AA women) [23]. This study data revealed that after adjusting for SES and the medical standard of care, the overall survival (OS) of women with TNBC was independent of their ethnicity [23]. Furthermore, a recent study on the neighborhood social determinants of TNBC indicated that the neighborhood disadvantage evaluated by the concentration disadvantage index was related to a more advanced stage of TNBC at diagnosis and worse stage-specific survival rates [24]. Notably, the TNBC incidence is higher among AA [24]. However, the concentration disadvantage index was still not able to explain certain disparities, indicating a possible impact of various genetic factors [24]. Hopefully, progress in the next-generation sequencing techniques will aid in conducting genome-wide association studies, which enables a more accurate determination of genetic factors that are responsible for TNBC disparities in various ethnic populations [25, 26]. Nevertheless, the impact of SES (e.g., poverty, illiteracy, and barriers to medical care access) in the AA population can considerably account for a worse prognosis and higher mortality rates among these patients [27].

## A glance at new and emerging targeted therapies for TNBC

The main difficulties in the treatment of patients with TNBC are caused by the tumor’s genetic diversity, TME heterogeneity, as well as a variety of metabolic, hormonal, reproductive, environmental, and SES variables. As a consequence, the absence of ER, PR, and HER2 expression eliminates certain treatment options for the locally advanced or metastatic TNBC, or reduces them to standard cytotoxic CHTs (e.g., anthracyclines, alkylating agents, and taxanes) [28]. This is very unfortunate, especially in difficultly managed TNBC cases, which are commonly encountered among AA women.

At present, some new targeted therapies have been approved by the Food and Drug Administration, including poly ADP-ribose polymerase (PARP) inhibitors (e.g., olaparib and talazoparib, for patients with *BRCA1/2* mutations), immune checkpoint inhibitors [e.g., atezolizumab-anti-programmed cell death ligand 1 (PD-L1) monoclonal antibody, and pembrolizumab—anti-programmed cell death protein-1 (PD-1) monoclonal antibody], and a combination of a monoclonal antibody targeting trophoblast cell-surface antigen 2 (Trop-2) with SN-38 (sacituzumab govitecan-hziy, an antibody-drug conjugate) (Table 1) [29–33]. Moreover, some emerging strategies which target tumor-driving signaling pathways in TNBC, including epidermal growth factor antibodies, phosphoinositide-3 kinase/protein kinase B/the mammalian target of rapamycin, angiogenesis inhibitors, androgen receptor antagonists, and estrogen receptor-beta agonists have been explored in clinical trials [34–38]. However, patients from various ethnic groups like AA should be more adequately represented in such ongoing and future studies in this field.

**Table 1.** Recently approved targeted therapies to treat patients with advanced or metastatic TNBC (PARP inhibitors, immune checkpoint inhibitors, and antibody-drug conjugate)

**Therapeutic target**	**Treatment class Drug/combination**	**RCT (phase 3) identifier**	**Therapeutic considerations**	**Author, year, reference**
ADP ribose polymerase PARP 1	PARP1 inhibitor olaparib	OlympiAD NCT02000622	In the OlympiAD study, olaparib has improved PFS compared with CHT of physician's choice, among pts with a g*BRCA1/2* mutation & HER2-negative metastatic BC	Robson et al. 2019 [29]
ADP ribose polymerase PARP	PARP inhibitor talazoparib	EMBRACA NCT01945775	In the EMBRACA trial, talazoparib prolonged PFS *vs.* CHT & improved PRO in g*BRCA1/2*-mutated ABC	Litton et al. 2020 [30]
PD-L1	Anti-PD-L1 monoclonal antibody atezolizumab + nab-paclitaxel (CHT)	IMpassion130 NCT02425891	In the 2nd interim OS analysis of the IMpassion130 trial, no significant difference in OS between the treatment groups in the ITT population was reported, but a clinically meaningful OS benefit with atezolizumab + nab-paclitaxel in pts with PD-L1 immune cell-positive disease was noted. In pts with PD-L1 immune cell-positive metastatic TNBC, atezolizumab + nab-paclitaxel is a valid therapeutic option	Schmid et al. 2020 [31]
PD-1	Anti-PD-1 monoclonal antibody pembrolizumab + CHT	KEYNOTE-355 NCT02819518	Pembrolizumab-CHT revealed a clinically meaning- ful improvement in PFS *vs.* placebo-CHT, in pts with metastatic TNBC (CPS ≥ 10); such results suggest that the addition of pembrolizumab to standard CHT for the 1-st-line treatment of metastatic TNBC is beneficial	Cortes et al. 2020 [32]
The human (Trop-2)	Antibody-drug conjugate sacituzumab govitecan-hziy [a combination of a monoclonal antibody, targeting Trop-2 with SN-38 (a cytotoxic, potent topoisomerase I inhibitor; a metabolite of irinotecan, conjugated to the antibody by a cleavable linker)]	IMMU-132-01 NCT01631552	Sacituzumab govitecan-hziy was associated with durable objective responses in pts with heavily pretreated metastatic TNBC	Bardia et al. 2019 [33]

ABC: advanced breast cancer; CPS: combined positive score; g: germline; ITT: intention-to-treat; PFS: progression-free survival; pts: patients; PRO: patient-reported outcomes; RCT: randomized controlled trial

## The significance of insulin-like growth factor-1 and IGF-1R signaling pathways in obesity and their possible correlations with TNBC

Obesity-related metabolic dysfunctions may contribute to cancer development, metastatic progression, recurrence, and treatment resistance via different signaling pathways, which are often relevant to insulin and its receptors [39]. In addition to its well-known metabolic functions, insulin stimulates the main pathways involved in cell survival and replication via binding to insulin receptors that activate signaling cascades (e.g., the insulin receptor isoform A acts as a cell proliferation factor and its elevated levels are associated with tumorigenesis in different malignancies, including BC) [39].

Moreover, insulin-like growth factor-1 (IGF-1) binds to the IGF-1R and promotes the malignant progression (e.g., the IGF-1/IGF-1R system stimulates the phosphatidyl-inositol-3 kinase/AKT serine/threonine kinase 1, protein kinase B, the mammalian target of rapamycin, and the mitogen-activated protein kinase pathways) [40]. In essence, changes in IGF-1/IGF-1R signaling and the abnormal IGF-1/IGF-1R expression are related to cell proliferation, anti-apoptotic, epithelial to mesenchymal transition, and migratory effects in various neoplastic tumors (e.g., increased IGF-1R expression and high IGF-1 blood levels are associated with an augmented BC risk and shorter survival of patients with TNBC compared to those with ER-positive BC) [40]. Also, according to a recent study, it was reported that in TNBC cells, the IGF-1/IGF-1R system activates the focal adhesion kinase signal transduction pathway (that regulates the nuclear accumulation of yes-associated protein) and the expression of its target genes [40]. In this way, the elevated IGF-1 or IGF-1R levels can correlate with TNBC progression and poor clinical outcomes in some women with TNBC [40]. However, further studies to explore the IGF-1/IGF-1R/focal adhesion kinase/yes-associated protein signaling pathways in TNBC are still needed [40].

Furthermore, based on recent study findings, the reduction of IGF-1R function increases cellular stress and cytokine production that, in turn, can alter TME [41]. It has been suggested that high expression of the IGF-1R is correlated with HR-positive BC and a more positive prognosis, while low expression of IGF-1R is associated with a negative prognosis in patients with TNBC [41]. Also, it has been shown that the IGF-1R plays a suppressive role for a primary tumor and metastatic lesions in TNBC (in the animal model) [41]. It is conceivable that the reduction of IGF-1R function increases cellular stress and cytokine production to promote an aggressive TME [41]. However, the mechanisms by which the reduced IGF-1R may contribute to adverse prognosis in TNBC remain unclear. Almost a half of BCs express the activated form of the IGF-1R [42]. Notably, AA women have higher IGF-1R expression in breast tissue, while EA women have higher levels of IGF-2R. Such a difference between the IGF-1R and IGF-2R expression may explain the higher rates of the more aggressive TNBC subtype among AA women compared to their EA counterparts [42, 43].

## A role of lipogenic enzymes (ATP-citrate lyase and fatty acid synthase) and their inhibitors as potential therapeutic targets in TNBC

Due to the scarcity of targeted therapies and the worst prognosis of TNBC, an intense search for innovative therapeutic approaches is merited, including some inhibitors of lipogenic enzymes. Lipid metabolism plays an important role in cancer cell survival and some enzymes which regulate the synthesis of fatty acids are often overexpressed. Inhibition of these enzymes may lead to an interrupted proliferation of malignant cells. In particular, blockers of the main enzymes that participate in the metabolism of FAs, such as ATP-citrate lyase (ACLY), fatty acid synthase (FAS), and acetyl-CoA carboxylase (ACC), are evaluated as possible antineoplastic therapies [44].

ACLY (the main enzyme in the conversion of citrate derived from glycolytic metabolism into acetyl-CoA) is overexpressed in many cancer cell lines that are stimulated by insulin, growth factors, and hyperglycemia. The inhibition of the ACLY produces cytotoxic effects via disrupting FA synthesis, and thus the inhibitors of ACLY enzyme may hold therapeutic hopes for patients with malignancies. However, the beneficial and toxic effects of ACLY inhibitors need to be investigated in-depth in future studies [44].

FAS [a complex cytosolic enzyme that catalyzes the final step of fatty acid (FA) biosynthesis] is commonly overexpressed in malignant cells (including BC) and is relevant to an increased risk of cancer recurrence [44]. Furthermore, some TME effects like hypoxia and acidity are critically important in the regulation of FAS [44]. FAS inhibition can block proliferation and induce the apoptosis of malignant cells with only minimal adverse effects for healthy cells [44]. For instance, orlistat (a pancreatic lipase blocker) is a FAS inhibitor that was originally designed for obesity treatment. In addition, orlistat revealed a decreased proliferation and increased apoptosis in HER2-positive BC. Unfortunately, due to its poor oral bioavailability and metabolic instability, the application of orlistat for anticancer therapy was not possible. However, the use of some novel drug-delivery systems based on nanoparticles may improve its bioavailability and decrease its toxic effects in women with aggressive BC subtypes in the future. For such reasons, FAS inhibitors have now been intensely explored in research studies [44].

ACC (the rate-limiting enzyme in FA synthesis) catalyzes the synthesis of malonyl-CoA and can be overexpressed in malignancies [44]. ACC inhibition is a target that can play a role in some antineoplastic therapies, but its metabolic effects need to be investigated in further clinical trials [44]. Considerations for targeting FA metabolism in cancer therapies require future research studies focused on the complex interplay between oncogenic signaling and dysregulated FA metabolism.

## Conclusion

Obesity, which poses a serious risk for many chronic diseases (e.g., DMT2, HTN, and CV disease), is also related to malignancy development (that occurs more frequently in AA women compared to EA women). It should be emphasized that obesity associated with abnormal metabolism and inflammation significantly increases the BC risk, incidence, prevalence, recurrence, and mortality rates, especially among AA patients.

In order to precisely explore possible relations between obesity and BC subtypes, it would be beneficial to use the combination of anthropomorphic measurements (e.g., BMI and WHR) and multiple metabolic, endocrine, and inflammatory parameters across different ethnic groups of women. Considering the rising obesity waves, especially in AA women as well as some ethnic differences in the metabolism, genetics, and the biology of TME, it is critical to know how the excessive body adiposity and relevant comorbidities affect different aspects of BC management and patient survival. This is critically important for an aggressive subtype of BC such as TNBC.

Importantly, in a process of transition from normal anthropometric and metabolic parameters to obesity associated with a dysfunctional metabolism, the inflammatory cytokines (e.g., IL-6) often influence a pro-carcinogenic transformation of the breast tissue and TME, contributing to the TNBC initiation or progression. Since obesity has a disproportionate impact on the TNBC subtype, it is imperative to spare no efforts to “turn the tide” against obesity, particularly in AA women.

In addition, it is critical to achieve and maintain a proper balance among the metabolic, hormonal, and immune system regulation, because it allows restriction of the inflammation to local areas. Such a balance permits the maintenance of lean adipocytes which secrete beneficial and anti-inflammatory cytokines. This, in turn, may slow down or even apprehend a devastating cascade during which the adipose tissue undergoes hypertrophy and secretes inflammatory cytokines that are related to the undesirable immune cell-shifting and immune system exhaustion.

Under these circumstances which often initiate or promote the malignant processes, the TNBC and its TME can display very aggressive behavior in the afflicted AA patients. Therefore, maintaining a healthy body weight, “flexible” metabolism, and a well-functioning immune system are of great importance.

As the global population continues to be more obese, the burden of BC will increase, as will the relevance of metabolic and immune health preservation. In this context, it is vital to conduct research and disseminate current knowledge about the intricate connections of the metabolic and immune systems, contributing to the TNBC aggressive course. Simultaneously, it is merited to design and implement feasible programs for patient education and support that would help “bridge the gap” between clinical research and practice, focusing on TNBC associated with obesity, metabolic comorbidities, and inflammation, particularly in vulnerable groups such as AA women. Further explorations are warranted to assess the multi-pronged factors that can affect TNBC prevention, management, and outcomes to optimize therapeutic approaches and survival among AA women.

## Abbreviations

AA: African American
ACC: acetyl-CoA carboxylase
ACLY: ATP-citrate lyase
AMBER: African American Breast Cancer Epidemiology and Risk
AR: androgen receptor
BC: breast cancer
BL: basal-like
BMI: body mass index
*BRCA*: breast cancer gene
CBCS: Carolina Breast Cancer Study
CHT: chemotherapy
CRP: C-reactive protein
CV: cardiovascular
DMT2: diabetes mellitus type 2
EA: European American
ER: estrogen receptor
FA: fatty acid
FAS: fatty acid synthase
HER2: human epidermal growth factor receptor 2
HR: hormone receptor
HTN: arterial hypertension
IGF-1: insulin-like growth factor-1
IGF-1R: insulin-like growth factor receptor type 1
IL-6: interleukin-6
INF-gamma: interferon-gamma
IR: insulin resistance
LAR: luminal androgen receptor
M: macrophage
MS: metabolic syndrome
OS: overall survival
*PALB2*: partner and localizer of breast cance gene 2
PARP: poly ADP-ribose polymerase
PD-1: programmed cell death protein-1
PD-L1: programmed cell death ligand 1
PFS: progression-free survival
PR: progesterone receptor
SES: socio-economic status
TME: tumor microenvironment
TNBC: triple-negative breast cancer
TNF-alpha: tumor necrosis factor-alpha
Trop-2: trophoblast cell-surface antigen 2
WC: waist circumference
WCHS: Women’s Circle of Health Study
WHR: waist-to-hip ratio

## Declarations

### Copyright

© The Author(s) 2021.
